# Whole-Brain Monosynaptic Afferent Projections to the Cholecystokinin Neurons of the Suprachiasmatic Nucleus

**DOI:** 10.3389/fnins.2018.00807

**Published:** 2018-11-05

**Authors:** Xiang-Shan Yuan, Hao-Hua Wei, Wei Xu, Lu Wang, Wei-Min Qu, Rui-Xi Li, Zhi-Li Huang

**Affiliations:** Department of Pharmacology, Department of Anatomy, School of Basic Medical Sciences, State Key Laboratory of Medical Neurobiology, Institutes of Brain Science and Collaborative Innovation Center for Brain Science, Fudan University, Shanghai, China

**Keywords:** cholecystokinin neuron, circadian rhythm, monosynaptic inputs, rabies viruses, suprachiasmatic nucleus

## Abstract

The suprachiasmatic nucleus (SCN) is the principal pacemaker driving the circadian rhythms of physiological behaviors. The SCN consists of distinct neurons expressing neuropeptides, including arginine vasopressin (AVP), vasoactive intestinal polypeptide (VIP), gastrin-releasing peptide (GRP), cholecystokinin (CCK), and so on. AVP, VIP, and GRP neurons receive light stimulation from the retina to synchronize endogenous circadian clocks with the solar day, whereas CCK neurons are not directly innervated by retinal ganglion cells and may be involved in the non-photic regulation of the circadian clock. To better understand the function of CCK neurons in non-photic circadian rhythm, it is vital to clarify the direct afferent inputs to CCK neurons in the SCN. Here, we utilized a recently developed rabies virus- and Cre/loxP-based, cell type-specific, retrograde tracing system to map and quantitatively analyze the whole-brain monosynaptic inputs to SCN CCK neurons. We found that SCN CCK neurons received direct inputs from 29 brain nuclei. Among these nuclei, paraventricular nucleus of the hypothalamus (PVH), paraventricular nucleus of the thalamus (PVT), supraoptic nucleus (SON), ventromedial nucleus of the hypothalamus, and seven other nuclei sent numerous inputs to CCK neurons. Moderate inputs originated from the zona incerta, periventricular hypothalamic nucleus, and five other nuclei. A few inputs to CCK neurons originated from the orbital frontal cortex, prelimbic cortex, cingulate cortex, claustrum, and seven other nuclei. In addition, SCN CCK neurons were preferentially innervated by AVP neurons of the ipsilateral PVH and SON rather than their contralateral counterpart, whereas the contralateral PVT sent more projections to CCK neurons than to its ipsilateral counterpart. Taken together, these results expand our knowledge of the specific innervation to mouse SCN CCK neurons and provide an important indication for further investigations on the function of CCK neurons.

## Introduction

The suprachiasmatic nucleus (SCN) has been widely known as a central pacemaker that orchestrates physiological and behavioral circadian rhythms that need daily synchronization to stay in phase with the 24-h solar cycle. Light information is transmitted to the SCN by the retinohypothalamic tract (RHT), which is a monosynaptic projection from the retina to the SCN (Pickard, 1982; Moore et al., 2002; Hannibal and Fahrenkrug, 2006). The SCN is a small heterogeneous structure that contains diverse subpopulations of neurons expressing distinct neuropeptides, including arginine vasopressin (AVP), vasoactive intestinal polypeptide (VIP), gastrin-releasing peptide (GRP), calretinin, cholecystokinin (CCK), and so on (Abrahamson and Moore, 2001; LeSauter et al., 2002; Moore et al., 2002; Hannibal et al., 2010). The AVP, VIP, and GRP neurons in the SCN have been elucidated as retinorecipient neurons that receive direct inputs from the RHT, indicating that these neurons play an important role in mediating the effects of light on the circadian clock (Abrahamson and Moore, 2001; Antle and Silver, 2005; Fernandez et al., 2016). The CCK neuron was also identified as a distinct cell type in the SCN (Morin, 2013), but it did not receive projections from the RHT and was not activated by light (Hannibal et al., 2010). The circadian rhythm is principally synchronized by light, but it can also be regulated by non-photic signals, such as exercise, feeding, and temperature (Buxton et al., 2003; Escobar et al., 2009; Buhr et al., 2010; Burgess et al., 2010; Morris et al., 2012). Thus, the literature suggests that SCN CCK neurons may be involved in the non-photic regulation of circadian rhythm. Previous studies have shown that feeding and temperature signals were mediated by the diencephalon, including the preoptic area, dorsomedial nucleus of the hypothalamus (DMH), arcuate nucleus of the hypothalamus (Arc), and paraventricular nucleus of the thalamus (PVT) (Tan et al., 2016; Hume et al., 2017; Zhao et al., 2017). However, it is not clear whether these nuclei send direct projections to CCK neurons of the SCN to entrain the circadian clock. Therefore, identifying the afferent circuits of SCN CCK neurons is critical to comprehensively understand the function of these neurons.

Traditional tracing approaches with non-specific tracers have successfully classified three major afferent inputs to the SCN: the RHT, geniculohypothalamic tract (GHT), and a pathway from the raphe nuclei (Hannibal and Fahrenkrug, 2006; Fernandez et al., 2016). However, a recent study used immunohistochemical staining to reveal that CCK neurons did not receive projections form these three major afferent pathways (Hannibal et al., 2010). The staining had obvious flaws in term of non-specificity and non-systematicity, making it difficult to assess the specific afferent distributions within the SCN. To overcome these limitations and detect all of the specific afferent inputs to SCN CCK neurons from the whole brain, it is necessary to adopt a specific, viral-mediated tracing system.

In recent years, a genetically modified rabies virus combined with Cre-LoxP technology, has been used to trace monosynaptic inputs (Wickersham et al., 2007; Lerner et al., 2015) and characterize the whole-brain presynaptic neurons of a specific neuron type within a complicated neural network (Do et al., 2016; Hu et al., 2016; Su et al., 2018). In our study, we utilized this viral tracing system to map the whole-brain afferent inputs to SCN CCK neurons. We found 29 afferent brain nuclei, including several important nuclei that integrated circadian, ingestive, and osmotic information to SCN CCK neurons. Our quantitative results provide numerous evidence for the structural framework of SCN CCK neurons and can guide further investigations of neuronal pathways that mediate functions of CCK neurons in the SCN.

## Materials and methods

### Animals

Pathogen-free, adult CCK-ires-Cre mice (Taniguchi et al., 2011) of either sex (10–12 weeks, 25–28 g) on a C57BL/6J background and wild-type littermates were used in these experiments. CCK-ires-Cre mice express Cre recombinase under the control of the CCK gene promoter. The animals were housed in individual cages at constant temperature (22 ± 0.5°C) and relative humidity (60 ± 2%) on an automatically controlled 12:12 light:dark cycle (lights on at 7 a.m.; 100 lux intensity) (Zhang et al., 2017) with free access to food and water. All of the animal studies were performed in accordance with protocols approved by the Committee on the Ethics of Animal Experiments of Fudan University Shanghai Medical College (permit number: 20110307-049). Every effort was made to minimize the number of animals used as well as any pain or discomfort experienced by the subjects.

### Viruses and surgery

AAV-CAG-DIO-TVA-GFP (Adeno-associated virus (AAV) 2/9 serotype; titer 1.7 × 10^13^ genomic copies/ml), AAV-CAG-DIO-RG (AAV2/9 serotype; titer 6.8 × 10^12^ genomic copies/ml), and EnvA-pseudotyped, glycoprotein (RG)-deleted, dsRed-expressing rabies virus (RV-EvnA-DG-dsRed; RV 5.0 × 10^8^ genomic copies/ml) were packaged and provided by BrainVTA (Wuhan, China). The detailed production and concentration procedures for the modified rabies virus were conducted as previously described (Pollak Dorocic et al., 2014; Hu et al., 2016).

Surgical procedures were carried out according to previous studies (Hu et al., 2016; Yuan et al., 2017). Briefly, naïve mice were anesthetized with chloral hydrate (360 mg/kg) and placed in a stereotaxic apparatus (RWD Life Science, Shenzhen, China). After exposing the skull and drilling a small hole, a glass micropipette was placed above the SCN [0.5 mm posterior and 0.2 mm lateral from bregma; 5.1 mm ventral from the pial surface]. First, two helper AAVs (AAV-CAG-DIO-TVA-GFP and AAV-CAG-DIO-RG were mixed at a 1:1 ratio in 50 nL) were injected into the SCN at 0.01 μL/min using a microsyringe pump controller (WPI, Sarasota, FL). To allow diffusion of the virus, the micropipette was not retracted until 15 min after the end of the injection. Three weeks later, 100 nL RV-EnvA-DG-dsRed was similarly injected into the same site. The scalp wound was closed with surgical sutures, and each mouse was kept in a warm environment until it resumed normal activity as previously described (Luo et al., 2018b).

### Single-cell RT-PCR

At 1–2 weeks after only helper AAV injections, CCK-Cre mice were anesthetized and perfused transcardially with ice-cold modified aCSF saturated with 95% O_2_ and 5% CO_2_ and containing (in mM): 215 sucrose, 26 NaHCO_3_, 10 glucose, 3 MgSO_4_, 2.5 KCl, 1.25 NaH_2_PO_4_, 0.6 mM Na-pyruvate, 0.4 ascorbic acid, and 0.1 CaCl_2_. Brains were then rapidly removed, and acute coronal slices (300 μm) containing the SCN were cut on a vibratome (VT1200, Leica) in ice-cold modified aCSF. Next, slices were transferred to a holding chamber containing normal recording aCSF (in mM): 125 NaCl, 26 NaHCO_3_, 25 glucose, 2.5 KCl, 2 CaCl_2_, 1.25 NaH_2_PO_4_ and 1.0 MgSO_4_, and allowed to recover for 30 min at 32°C. Then, slices were maintained at room temperature (RT) for 30 min before recording.

During recording, slices were submerged in a recording chamber superfused with aCSF (2 mL/min) at 30–32°C. Slices were visualized using a fixed-stage upright microscope (BX51W1, Olympus, Japan) equipped with a 40 × water immersion objective and an infrared-sensitive CCD camera. The CCK neurons were identified based on their GFP expression in the SCN and the cytosolic content was aspirated into the patch pipette, and expelled into a 200 μL PCR tube as described previously (Luo et al., 2018b). The single-cell reverse-transcription PCR (RT-PCR) protocol was designed to detect the presence of mRNA coding for CCK. Reverse transcription and PCR amplification were performed with gene-specific multiplex primer using the SuperScript III One-Step RT-PCR kit (catalog number: 12574018, ThermoFisher). The reaction was performed as follows: 30 min at 55°C, 2 min at 94°C; 70 cycles of 20 s at 94°C, 30 s at 61°C, and 25 s at 68°C; and 5 min at 68°C. The PCR products were visualized by electrophoresis in agarose gels (1.5%) with ethidium bromide. The expected size of each final PCR product is CCK 215 bp. The specific primers for CCK gene were custom designed and synthesized (Biosune Biotechnology, Shanghai). CCK-F primer, 5′ to 3′: AAGCCATGAAGAGCGGCGTAT; CCK-R primer, 5′ to 3′: GCGGACCTGCTGGATGTATCG.

### Histology and image analysis

One week after injection of the rabies virus, mice were perfused with 10 mL saline, followed by 100 mL of 4% paraformaldehyde in 0.1 M phosphate buffer (PB, pH 7.4). The brains were removed, post-fixed for 4–6 h at 4°C, and then cryoprotected in 10, 20, and 30% sucrose in 0.1 M PB at 4°C until they sank. Tissues were embedded in OCT compound, and stored at −80°C before use. The brains were coronally sectioned at a thickness of 30 μm on a cryostat (Leica 1950) in three series and were collected in 0.01 M phosphate-buffered saline (PBS, pH 7.4). Every third section was counterstained with DAPI (1:3000, Sigma-Aldrich, USA). The stained sections were then coverslipped with Fluoromount-G™ (Southern Biotech).

Images of whole-brain sections were captured using a 20 × objective on an Olympus microscope (Olympus VS-120, Tokyo, Japan). Further imaging analyses were performed using Olympus analysis software and ImageJ software. The number of afferent neurons and area of each input regions were counted by ImageJ automatically, and we normalized the data in every input region. Quantification of the subregion boundaries was based on the mouse brain atlas of Paxinos and Franklin (Paxinos and Franklin, 2001). The proportion of cell in each nucleus was calculated as the ratio of the normalized number of dsRed-labeled cells in each nucleus to the total number of dsRed-labeled cells, and the cell density was defined as the number of dsRed-labeled cells per unit area within each nucleus. According to the proportion of cells in each nucleus, we defined three grades of afferent inputs as numerous input (over 4%), moderate input (1–4%), and a few input (<1%).

To characterize the inputs from the paraventricular nucleus of the hypothalamus (PVH), and supraoptic nucleus (SON), we immunostained the brain slices containing the two areas with AVP and oxytocin (Oxt) antibodies according to the following protocol (Yuan et al., 2017). Brain sections from the PVH and SON were incubated overnight at 4°C in PBS containing 5% normal donkey serum (v/v), 0.3% Triton X-100 (v/v), and the following primary antibodies: rabbit anti-AVP (1:2000, catalog number: 20069, Immunostar) and rabbit anti-Oxt (1:2000, catalog number: ab212193, Abcam). After several washes in PBS, the sections were incubated with Alexa Fluor-conjugated IgG antibody (Invitrogen) at room temperature for 2 h. The sections were then incubated in PBS containing DAPI. Finally, sections were coverslipped with Fluoromount-G™ (Southern Biotech). Fluorescence images were collected using a Leica confocal system.

## Results

### Mapping monosynaptic inputs onto SCN CCK neurons using a rabies-based tracing system

**Figure 1:**
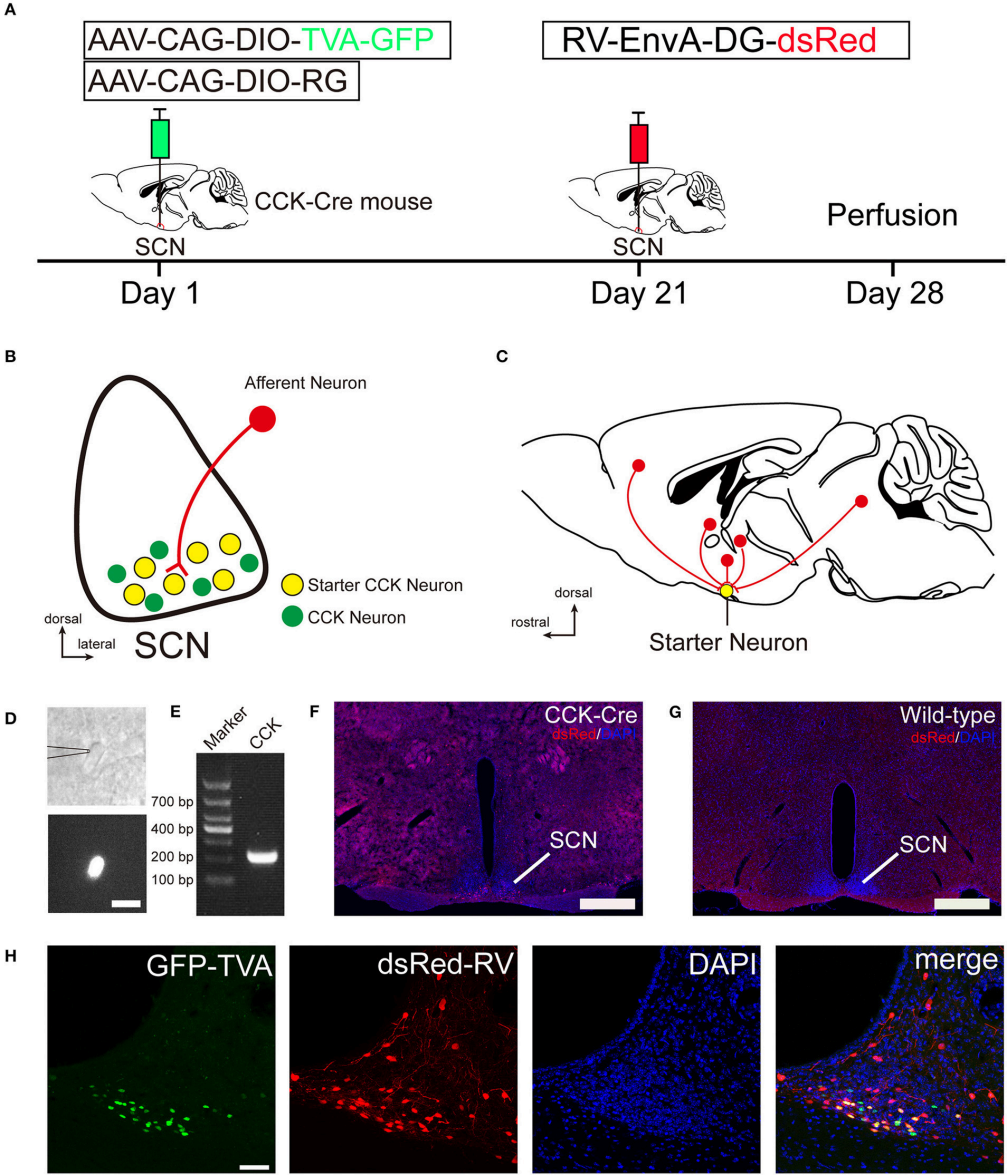
Monosynaptic afferent tracing on SCN CCK neurons with a rabies virus-based, retrograde tracing system. **(A)** Design of viral vectors for RV-mediated trans-synaptic retrograde tracing and experimental timeline for unilateral injections of AAV and RV in the SCN of CCK-Cre mice. **(B)** Schematic illustration of the starter neuron (yellow, **B**) after AAV helper virus (green) and rabies virus (red) injection into SCN CCK neurons. **(C)** Schematic illustration of whole-brain, monosynaptic input (red) to CCK starter neurons (yellow). **(D)** A typical section of an CCK-Cre mouse injected with helper AAVs into the SCN for patch-clamp electrophysiology shows an GFP-expressing neuron for recording, the patch pipette attached to the membrane of the recorded neuron in phase contrast (upper panel), and the recorded neuron with fluorescent contrast (lower panel). Scale bar, 15 μm. **(E)** Representative result from a single-cell RT-PCR reaction confirming the CCK phenotype in the GFP labeled neuron of the SCN. **(F,G)** Fluorescence images showing RV-labeled neurons (red) in CCK-Cre mice **(F)**, but not in wild-type mice **(G)**. Scale bar, 500 μm. **(H)** Fluorescence images showing that starter neurons (yellow) infected with AAV helper virus and RV were restricted to the unilateral SCN. Scale bar, 50 μm. Data were obtained from independent experiments.

To identify the monosynaptic afferent inputs to SCN CCK neurons, we used rabies virus-mediated, trans-synaptic, retrograde tracing on a transgenic mouse line expressing Cre recombinase in CCK neurons (Taniguchi et al., 2011). This retrograde, viral tracing system has been shown to label monosynaptic inputs to the desired starter cells with high specificity (Lerner et al., 2015; Do et al., 2016; Hu et al., 2016). In CCK-Cre mice, we applied a genetically engineered viral system to map the whole-brain afferent inputs to SCN CCK neurons. We first co-expressed the avian receptor TVA and the rabies glycoprotein G (RG) in SCN CCK neurons, which was achieved by a unilateral injection of two AAV-DIO helper viruses (AAV-CAG-DIO-TVA-GFP and AAV-CAG-DIO-RG) into the SCN (Figure 1A) of CCK-Cre mice. Three weeks later, RV-EnvA-DG-dsRed was injected into the same location, where it only infected cells expressing TVA and required RG to spread retrogradely into presynaptic cells (Figures 1A–C). After 1 week, the starter neurons could be characterized by the co-expression of RV-EnvA-DG-dsRed and AAV-CAG-DIO-TVA-GFP. We used single-cell RT-PCR to detect the presence of CCK mRNA in GFP-positive neurons in the SCN (7 of 7, *n* = 3 CCK-Cre mice), and confirmed that these neurons are CCK-expressing neurons (Figures 1D,E). We found that the starter neurons were restricted to the ventral part of the rostral and middle SCN, ipsilateral to the injection site (Figures 1F,H). Moreover, we observed numerous neurons in the SCN that were dsRed-positive but did not express GFP, demonstrating the presence of direct, monosynaptic input from other types of SCN neurons to the CCK neurons (Figure 1H). Upon conducting the same injection protocol in naïve animals, we did not detect the expression of GFP or dsRed in these wild-type mice that did not express Cre recombinase (Figure 1G). Thus, this technique could be used to map the whole-brain, monosynaptic afferent inputs to SCN CCK neurons (Figure 1C).

### SCN CCK neurons received direct inputs from 29 brain nuclei

**Figure 2:**
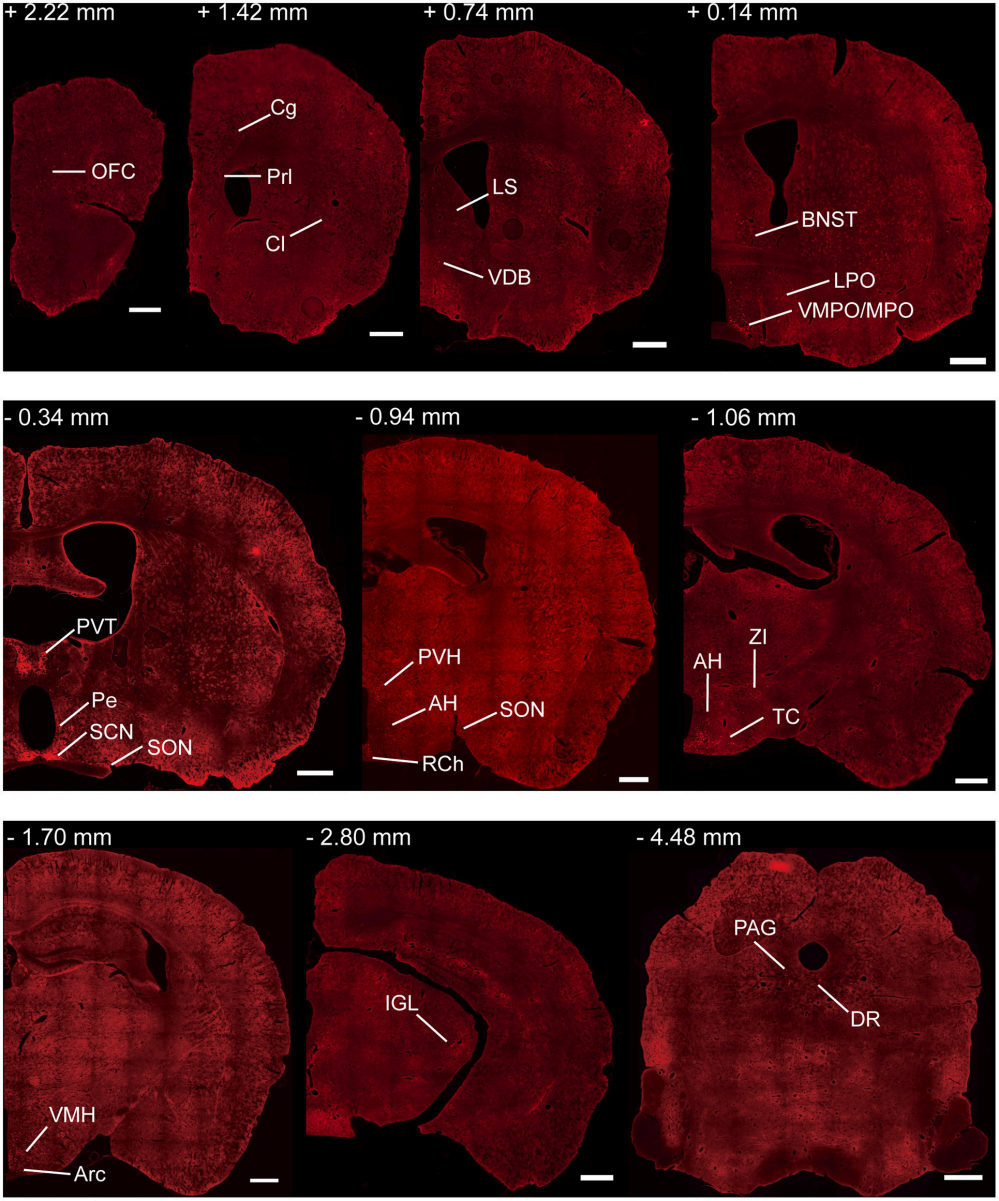
Representative coronal sections showing labeling of monosynaptic inputs to SCN CCK neurons. For some sections, only one hemisphere is shown. Scale bar, 500 μm. Data were obtained from four independent experiments. Abbreviations of the brain regions used are the following: AH, anterior hypothalamic area; Arc, arcuate nucleus of the hypothalamus; BNST, bed nucleus of stria terminalis; Cg, cingulate cortex; Cl, claustrum; DR, dorsal raphe nucleus; IGL, intergeniculate leaf; LPO, lateral preoptic nucleus; LS, lateral septum; MPO, medial preoptic nucleus; PAG, periaqueductal gray; Pe, periventricular nucleus of the hypothalamus; Prl, prelimbic cortex; PVH, paraventricular nucleus of the hypothalamus; PVT, paraventricular nucleus of the hypothalamus; RCh, retrochiasmatic area; SCN, suprachiasmatic nucleus; SON, supraoptic nucleus; TC, tuber cinereum area; VDB, nucleus of the vertical limb of the diagonal band; VMH, ventromedial nucleus of the hypothalamus; VMPO, ventromedial preoptic nucleus; ZI, zona incerta.

**Figure 3:**
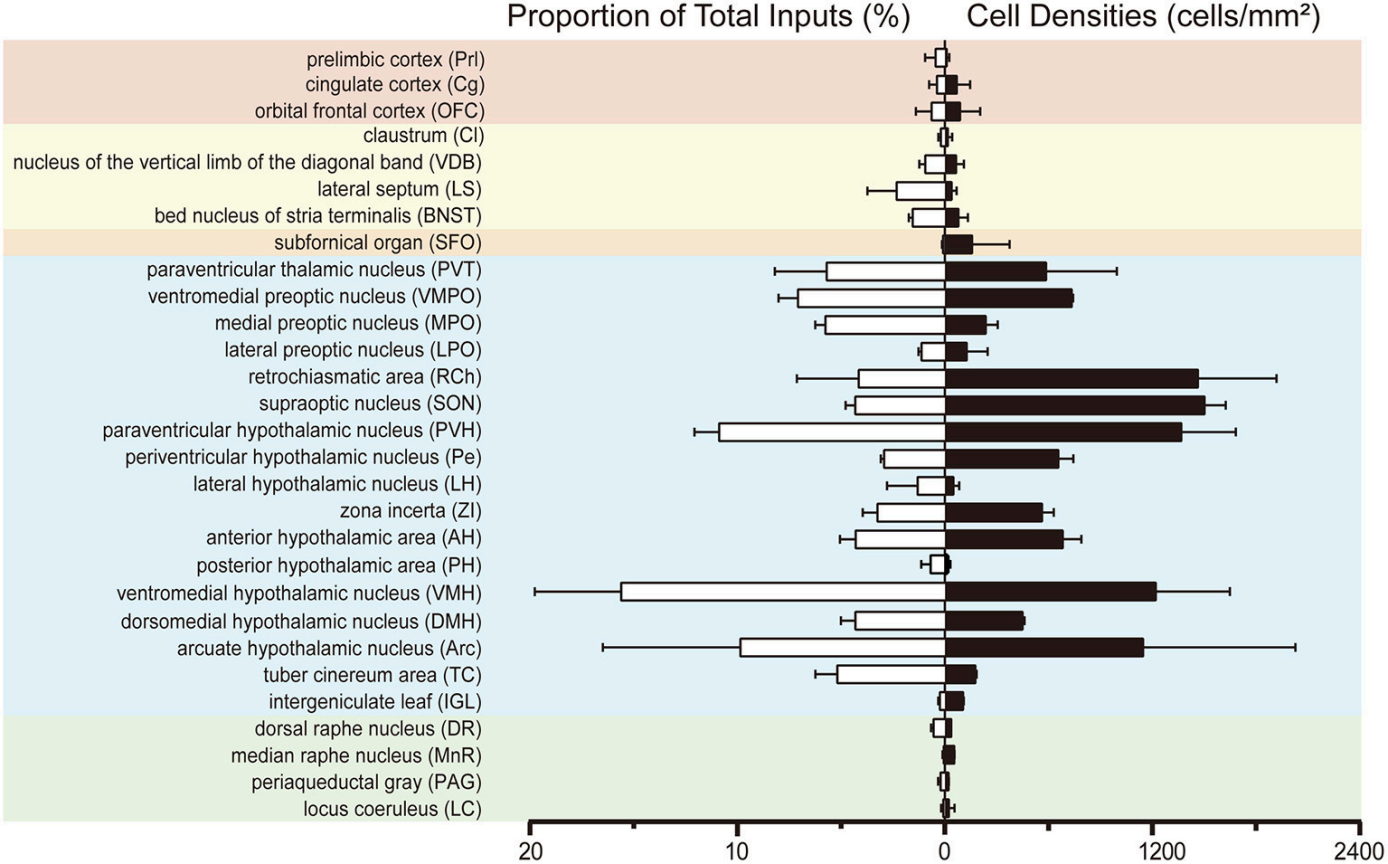
Statistical analysis of whole-brain, monosynaptic inputs to SCN CCK neurons. Normalized ratio **(Left)** of cell number in each input region against the total number of whole-brain inputs. Cell densities **(Right)** of monosynaptic inputs in each brain area. Error bar represents the SEM (*n* = 4 CCK-Cre mice).

**Figure 4:**
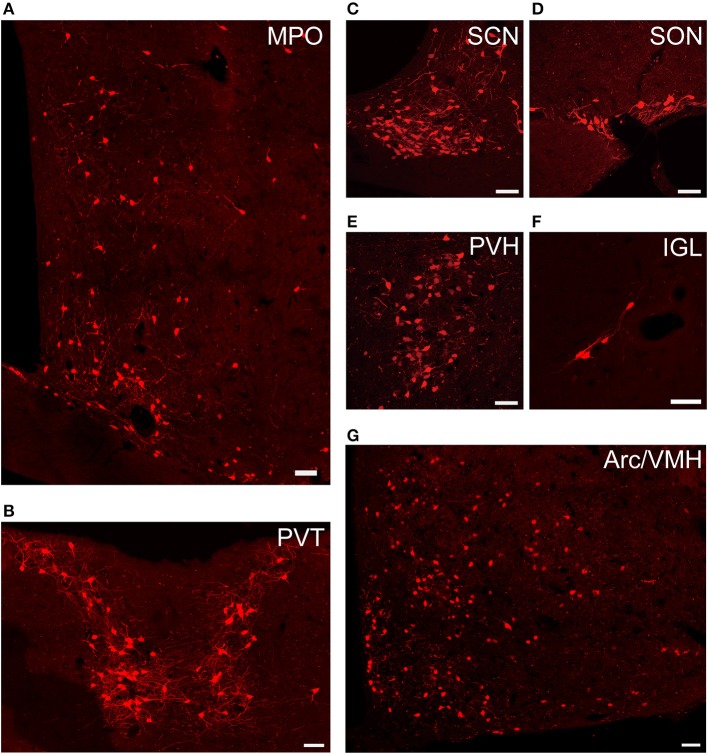
Representative images of selected regions with monosynaptic inputs to SCN CCK neurons. Data were obtained from four independent experiments. **(A)** MPO; **(B)** PVT; **(C)** SCN; **(D)** SON; **(E)** PVH; **(F)** IGL; **(G)** Arc, and VMH. Scale bar, 50 μm.

To investigate the whole-brain monosynaptic input areas to SCN CCK neurons, we examined the serial coronal whole-brain sections (Figure 2). In CCK-Cre mice injected with the three viruses, those neurons expressing only dsRed were the monosynaptic inputs to SCN CCK neurons. The dsRed-labeled presynaptic neurons were observed in 29 brain nuclei throughout the telencephalon, diencephalon, and brainstem (Figure 2). Furthermore, we measured the number of labeled neurons and the labeling density in individual brain areas to more quantitatively describe the whole-brain distribution of afferent input to SCN CCK neurons. The locations of labeled neurons were determined using a standard mouse brain atlas (Paxinos and Franklin, 2001). To correct potential bias, the number of dsRed-labeled cells in each nucleus was further normalized by the number of starter neurons. A list of whole-brain inputs was generated for SCN CCK neurons (Figure 3), which consisted of 29 different nuclei. Among these, CCK neurons received numerous afferent projections from 11 nuclei: PVH, PVT, SON, ventromedial nucleus of the hypothalamus (VMH), Arc, ventromedial preoptic nucleus (VMPO), medial preoptic nucleus (MPO), tuber cinereum area (TC), DMH, anterior hypothalamic area (AH), retrochiasmatic area (RCh). In addition, we found seven nuclei with moderate input nuclei contained the zona incerta, periventricular nucleus of the hypothalamus, lateral septum (LS), bed nucleus of stria terminalis (BNST), lateral hypothalamus (LH), lateral preoptic nucleus (LPO), and the nucleus of the vertical limb of the diagonal band (VDB). Finally, we observed 11 a few inputs to CCK neurons, which originated from the orbital frontal cortex (OFC), prelimbic cortex (Prl), cingulate cortex (Cg), claustrum, posterior hypothalamic area (PH), dorsal raphe nucleus (DR), intergeniculate leaf (IGL), periaqueductal gray (PAG), locus coeruleus (LC), subfornical organ (SFO), and median raphe nucleus (MnR). Although most of the dsRed-labeled presynaptic neurons were located in the hemisphere ipsilateral to the starter neurons, the contralateral PVT had stronger projections to SCN CCK neurons than the ipsilateral PVT (712.4 ± 565.9 vs. 410.5 ± 304.5 cells/mm^2^, Figure 4B). Furthermore, in comparison with previous studies, our study revealed five novel projections to CCK neurons, namely, the VDB, LPO, Prl, OFC, and Cg (Supplemental Figure 1) (Moga and Moore, 1997; Krout et al., 2002).

### Preferential inputs to SCN CCK neurons were from diencephalon, not from the three major SCN afferent pathways

We found that SCN CCK neurons integrated the majority of their inputs (91.97%) from the diencephalon, specifically from the VMH (16.47%), PVH (11.78%), Arc (9.37%), VMPO (7.66%), PVT (6.16%), MPO (6.23%), and SON (4.67%) (Figures 3, 4). Although the VMH provided the largest number of inputs to CCK neurons, the SON was the most densely labeled area (Figure 3). Interestingly, we detected no dsRed-labeled neurons in both ipsilateral and contralateral retina (data not shown), indicating that SCN CCK neurons did not receive direct input from the major photic input pathway, the RHT (Morin, 2013). Furthermore, we observed only sparse dsRed labeling in the IGL (0.26%) and MnR (0.05%) (Figures 3, 4F), which are part of the GHT and a serotonin pathway from the median raphe nuclei, respectively. In contrast with our results, these two major pathways were previously shown to send robust afferent inputs to the SCN (Morin, 2013). Our results also differed from the findings of Hannibal and colleagues, in which immunostaining did not reveal any direct innervation from the IGL and raphe nucleus to the CCK neurons of the SCN (Hannibal et al., 2010).

### SCN CCK neurons received inputs from AVP neurons in the PVH and SON

**Figure 5:**
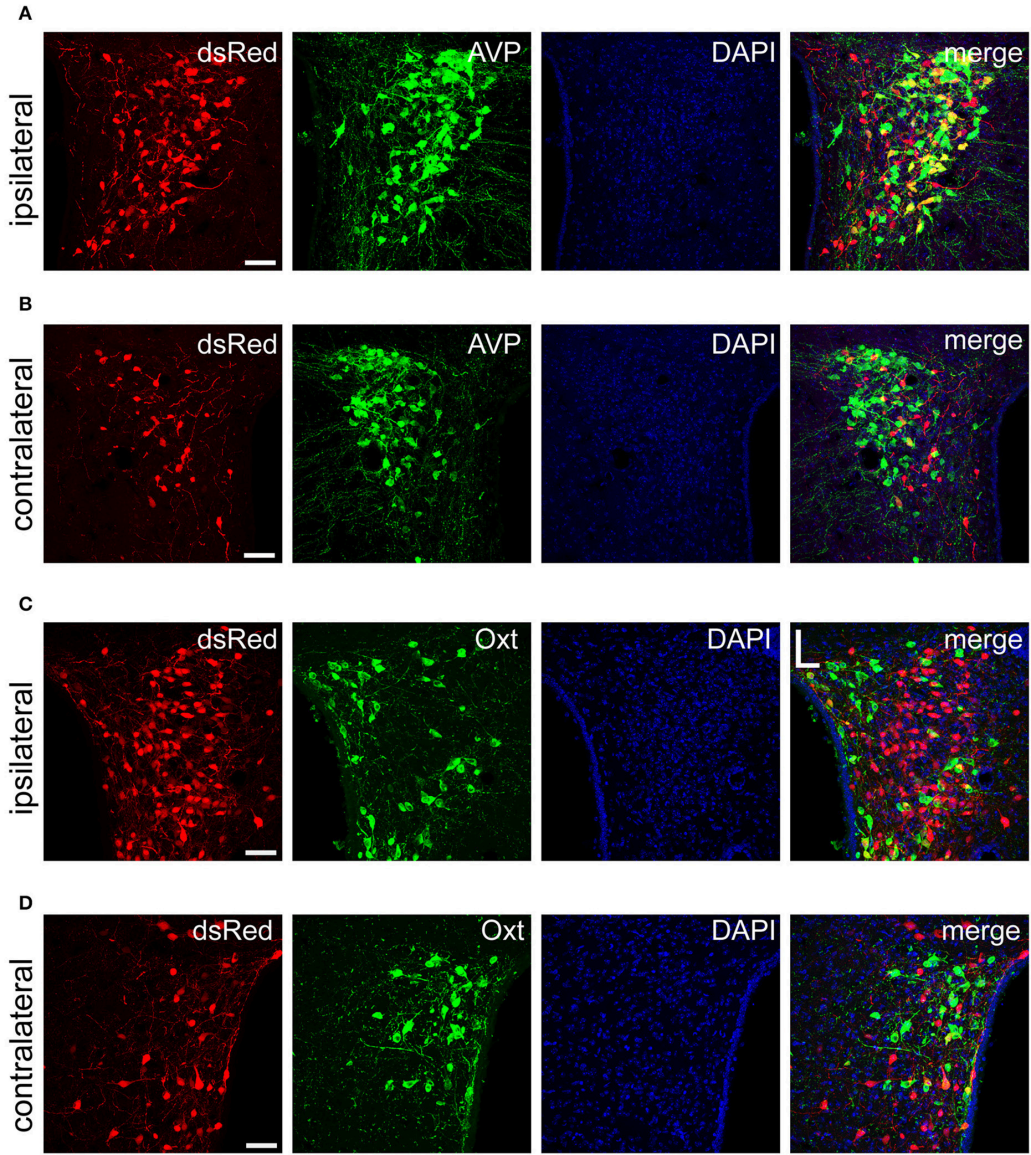
Immunofluorescence images showing dsRed-labeled afferent neuron with AVP and Oxt in the bilateral PVH. Immunofluorescence images showing that some dsRed-labeled neurons were co-localized with AVP neurons in the ipsilateral **(A)** and the contralateral PVH **(B)**. Immunofluorescence images showing that dsRed-labeled neurons rarely co-localized with Oxt neurons in the ipsilateral **(C)** and the contralateral PVH **(D)**. Scale bar, 50 μm. Data were obtained from four independent experiments.

**Figure 6:**
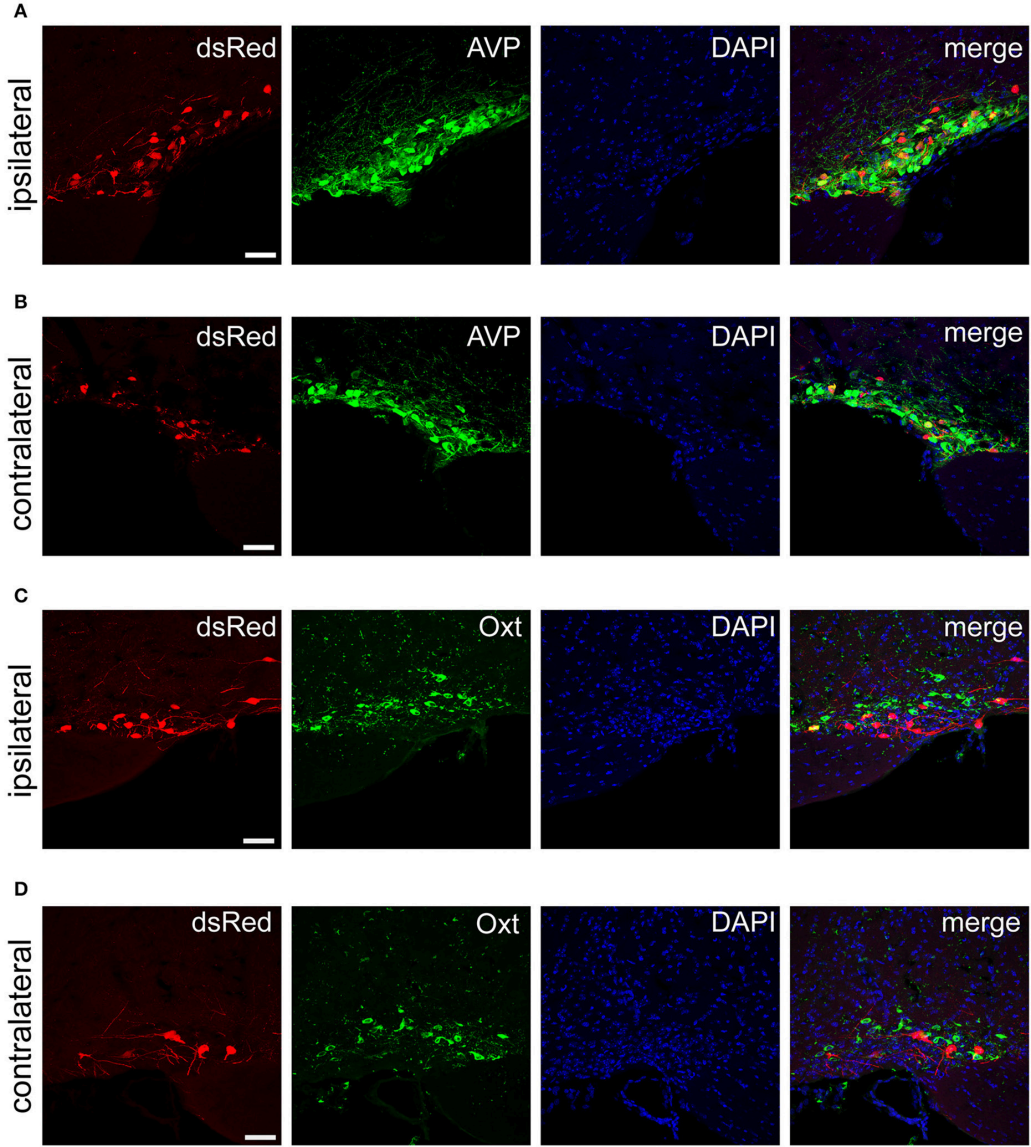
Immunofluorescence images showing dsRed-labeled afferent neurons with AVP and Oxt in the bilateral SON. Immunofluorescence images showing that some dsRed-labeled neurons were co-localized with AVP neurons in the ipsilateral **(A)** and the contralateral SON **(B)**. Immunofluorescence images showing that dsRed-labeled neurons rarely co-localized with Oxt neurons in the ipsilateral **(C)** and the contralateral SON **(D)**. Scale bar, 50 μm. Data were obtained from four independent experiments.

The PVH and SON of the hypothalamus are two important integrative brain structures that coordinate responses to perturbations in water balance and regulate maternal physiology through the release of the neuropeptide hormones AVP and Oxt (Qiu et al., 2011). In this study, we found that while SCN CCK neurons received significant afferent inputs from bilateral PVH (Figures 2, 4E), the ipsilateral PVH had more projections to SCN CCK neurons than the contralateral PVH (2058.0 ± 418.4 vs. 841.3 ± 341.0 cells/mm^2^, *n* = 4, Figures 5A,B). Immunostaining for AVP showed that many PVH AVP neurons sent direct inputs onto SCN CCK neurons, whereas Oxt immunostaining demonstrated that few PVH Oxt neurons sent direct projections to CCK neurons (Figures 5C,D). Moreover, our results revealed that bilateral SON sent direct projections to the CCK neurons in the SCN, with more monosynaptic inputs from the ipsilateral SON than the contralateral SON (2598.7 ± 369.5 vs. 645.6 ± 104.1 cells/mm^2^, *n* = 4), similar to the afferent pattern from the PVH. The immunostaining data for AVP and Oxt in the SON showed that many SON AVP neurons sent direct inputs onto SCN CCK neurons, whereas few SON Oxt neurons sent direct projections to SCN CCK neurons (Figures 6A–D). These results can provide a foundations for further investigations on links between AVP- and Oxt-mediated ingestive behavior or osmotic stability and circadian rhythm in the SCN.

## Discussion

To understand how SCN CCK neurons modulate non-photic behaviors, it is crucial to explore the afferent inputs that influence the activity of CCK neurons. In the present study, we clarified the whole-brain, direct, monosynaptic inputs to CCK neurons in the SCN using cell-type specific infection and retrograde spread of a modified rabies virus. We efficiently characterized the distribution of whole-brain input to SCN CCK neurons, which preferentially originated from a wide range of nuclei in the diencephalon, such as the VMH, Arc, MPO, PVT, PVH, SON, TC, and DMH. Moreover, the afferent pattern to CCK neurons in the SCN was strongly ipsilateral, with few contralateral projections other than the PVT. In addition, our results revealed specific inputs to the SCN and provided a comprehensive map of the presynaptic patterns that may control SCN CCK neuron activity.

### Comparison between specific trans-synaptic tracing and traditional tracing

The neural connectivity of the SCN has been extensively investigated due to its critical role in the circadian rhythm. Previous investigations used conventional tracing techniques, such as non-specific tracers, multisynaptic pseudorabies viruses, and immunohistochemistry (Moga and Moore, 1997; Abrahamson and Moore, 2001; Krout et al., 2002; Morin, 2013; Fernandez et al., 2016). Previous tract-tracing studies have consistently revealed that the SCN received input from three major pathways: direct visual input from the retina through the RHT, secondary visual input from the IGL of the lateral geniculate complex through the GHT, and a pathway from the midbrain raphe nuclei (Hannibal and Fahrenkrug, 2006). However, these tracing methods do not allow for the identification of specific afferents of SCN CCK neurons. Using the rabies virus-based approach of trans-synaptic retrograde tracing, we found that the retina did not provide direct input to SCN CCK neurons. These results can explain why CCK neurons were not light-responsive as evaluated by the induction of c-Fos (Hannibal et al., 2010). Moreover, we revealed that SCN CCK neurons received minor projections from the IGL and raphe nuclei, unlike previous reports showing robust input projections from these brain nuclei (Krout et al., 2002) or no inputs at all (Hannibal and Fahrenkrug, 2006). In addition, we detected retrograde labeling in several brain areas, namely the Prl, OFC, Cg, VDB, and LPO, which had not been previously identified as SCN inputs. These novel observations were likely due to the greater sensitivity of the trans-synaptic tracing method using the modified rabies virus. Traditional tract-tracing studies tended to inject small amounts of tracer into only a part of the SCN, in an attempt to avoid the potential confounding factors of tracer spillover and tracer pickup by fibers-of-passage. Different research groups have reported inconsistent labeling patterns, especially from brain areas that contributed a few and moderate inputs to the SCN (Moga and Moore, 1997; Krout et al., 2002). Using the more precise and efficient viral-mediated tracing method, our findings should provide a comprehensive map of the presynaptic patterns that control SCN CCK neurons.

### Implications for SCN CCK neurons in non-photic circadian rhythm

The SCN is widely considered to be the master circadian pacemaker necessary for physiological behaviors. Although light is the most potent zeitgeber to this master circadian oscillator, circadian clocks are entrained by both photic and non-photic signals. Previous studies found that AVP, VIP, and GRP neurons in the SCN received direct input from the retina, as well as indirect photic input from the IGL to integrate the non-image-forming photic information for circadian photo-entrainment (Abrahamson and Moore, 2001; Fernandez et al., 2016). As mentioned above, CCK neurons did not receive projections from the retina and were not directly entrained by light. However, our results revealed that there were robust dsRed-labeled neurons in the SCN, which did not co-express TVA-GFP, indicating that SCN CCK neurons received local innervations from intrinsic neurons in the SCN. It has been reported that SCN CCK neurons were innervated by the processes of SCN VIP neurons in mice and received contacts from SCN calbindin neurons in the ventral part of the central SCN in hamster (LeSauter et al., 2002, 2009; Hannibal et al., 2010). Therefore, SCN CCK neurons may relay optic or circadian signals from the VIP and calbindin neurons in the SCN and transmit these signals to mediate clock information.

Circadian entrainment can also occur in the absence of light, suggesting that non-photic signals can phase-shift and synchronize circadian clocks (Morris et al., 2012). Such non-photic signals include exercise, feeding, and temperature (McArthur et al., 1991; Damiola et al., 2000; Buxton et al., 2003; Escobar et al., 2009; Buhr et al., 2010). Previous findings showed that a majority of the preoptic neurons were activated by heat exposure and were located in the VMPO (Tan et al., 2016; Abbott and Saper, 2018). Our results revealed that the VMPO region had a robust projection to SCN CCK neurons; the interconnection between the two areas supports the idea of circadian entrainment by temperature. Moreover, hypothalamic nuclei, including PVH, Arc, DMH, VMH, and the lateral hypothalamus, were shown to govern energy balance via both metabolic and behavioral responses (Myers and Olson, 2012). Particularly, the VMH and Arc were thought to play a role in controlling food intake and peripheral metabolism (Dhillon et al., 2006; Zhang and van den Pol, 2016). Here, we revealed that SCN CCK neurons received numerous afferent inputs from the PVH, VMH, and Arc; thus, our results may provide a circuit-based explanation of how feeding can entrain circadian rhythm via SCN CCK neurons.

### Implications for SCN CCK neurons in ingestive behavior

Ingestive behavior in a natural environment is essential for survival, and is dependent on hunger signals, which involve interoceptive sensory neurons that monitor metabolic level and consequently regulate food-seeking and consumption behaviors (Trivedi, 2014; Cheng et al., 2018). Previous research has revealed many nuclei in the diencephalon as important sites that respond to feed restriction and regulate of ingestive behaviors, including the PVT, PVH, Arc, and VMH (Dhillon et al., 2006; Atasoy et al., 2012; Zhang and van den Pol, 2016; Jarvie et al., 2017; Ong et al., 2017; Luo et al., 2018a). Food deprivation incresed the synthesis of neuropeptide Y, one of the most potent orexigenic peptides, in neurons of the Arc (Sahu et al., 1988; Beck et al., 1992), and acute and chronic calorie restriction increased the activity of Arc neurons (Jarvie et al., 2017). Moreover, neurons in the VMH were inhibited by food deprivation (Kosta et al., 1987; Sternson et al., 2005; Flanagan-Cato et al., 2008). For the PVT, the effect of food deprivation are controversial. Nakahara et al. (2004) observed a remarkable increase in c-Fos expression in the PVT after restricted feeding for 2 h, suggesting increased neuronal activity by food deprivation. However, Zhang and van den Pol (2017) showed increased activity of ZI GABA neurons after food deprivation for 24 h. Since ZI GABA neurons inhibit PVT glutamatergic neurons, the findings by Zhang and van den Pol suggest that the activity of PVT neurons was inhibited by food deprivation. Together, these data indicate that feed restriction can change the activity of neurons in the Arc, VMH, and PVT. The Arc receives the hunger signals via the neural circuits that integrate visceral signals of energetic state and consequently regulate physiology and behavior via efferent outputs of the Arc neurons, consisting of the agouti-related peptide (AGRP) neurons and pro-opiomelanocortin neurons. Our results showed that the Arc had abundant projections to SCN CCK neurons; this finding is consistent with previous investigations using non-specific retrograde tracers that also showed that the SCN received major afferent inputs from the Arc. It has been speculated that the signal inputs from the Arc to CCK neurons could explain the circadian rhythm of feeding behavior, because CCK knockout mice eat more food than control animals during the light period and less food during the dark period (Lo et al., 2008). In addition, increasing evidence has shown that the PVT played a pivotal role in integrating information related to appetitive motivation, feeding, aversion, and anxiety (Hsu et al., 2014; Millan et al., 2017; Cheng et al., 2018). Our results, showing monosynaptic input from the PVT to SCN CCK neurons, suggest that CCK neurons could be involved in energy metabolism and anxiety-like behavior (Lo et al., 2008). Interestingly, we observed a preferential input from the contralateral PVT to CCK neurons, in contrast to the primarily ipsilateral pattern of innervation from the Arc, PVH, and VMH. The function of this contralateral-dominant innervation pattern between SCN CCK neurons and the PVT needs to be further studied.

### Implications for SCN CCK neurons in osmotic stability

Systemic osmoregulation is a vital homeostatic process because acute deviations in extracellular fluid osmolality can cause significant cellular shrinking or swelling and subsequent tissue and organ damage (Bedford and Leader, 1993). Thus, osmotic stability is controlled by centrally-mediated adjustments in the release of AVP from the hypothalamo-neurohypophyseal system. AVP is synthesized in the somata of magnocellular neurosecretory neurons located in the SON and PVH, the crucial integrative brain structures that coordinate responses to perturbations in water balance (Bargmann, 1966; Qiu et al., 2011). It has been reported that the firing activity of neurons in the PVH and SON was enhanced during water deprivation (Reis et al., 2012). Moreover, Landgraf and Ludwig (1991) have reported an increase in AVP release within the PVH and SON in response to local hypertonic artificial cerebrospinal fluid delivery. In addition, Miyata et al. (1994) have observed that the soma size of both AVP neurons in the SON in rat was enlarged in chronic osmotic stimulation. These data suggest that neurons in the PVH and SON, AVP-positive neurons, show response to osmotic disturbance.

In addition, in many species of mammals (Forsling, 2000; Moon et al., 2004), a progressive increase in circulating AVP concentration was observed during the sleep period and a trough around the wake period. This circadian rhythm in AVP levels is functionally important, because the absence of an AVP surge during the late-sleep stage resulted in polyuria and disrupted sleep (Miller, 2000). Recent findings revealed that clock neurons in the SCN sent direct, functional axonal projections to modulate the strength of the connection between the organum vasculosum lamina terminalis (the central osmosensory nucleus) and AVP neurons in the SON and PVH. This indicates that clock neurons can modulate circadian changes in osmotic and AVP regulation (Cui et al., 1997; Abrahamson and Moore, 2001; Kalsbeek et al., 2006; Trudel and Bourque, 2010, 2012). Our results suggest that SCN CCK neurons receive the osmotic signals from AVP neurons in the SON and PVH and may integrate the information of osmotic regulation with the principal pacemaker to generate the strongly reciprocal feedback circuit. When suffering from dehydration, this feedback circuit can help maintain circulating AVP at a predetermined level to exclude circadian fluctuations.

Notably, in addition to AVP expressing neurons, the PVH also contain another neuron populations, such as Oxt neurons in the magnocellular neuroendocrine group, neurons that synthesize and release corticotropin-releasing hormone, thyrotropinreleasing hormone, dopamine, somatostatin, or growth hormone-releasing hormone in the parvicellular neuroendocrine group, and neurons that project to the brainstem and spinal cord for the central autonomic regulation in the descending group (Kombian et al., 2002). In total, more than 30 neurotransmitters have been localized to neurons within the PVH (Pyner, 2009). Here, we found that a part of non-AVP- and non-Oxt-positive neurons in the PVH also sent direct inputs to SCN CCK neurons, suggesting that SCN CCK neurons may have other functions in addition to osmotic regulation.

Together, our viral tracing results provide a whole-brain map of neurons that convey reinforcement signals to SCN CCK neurons. Our findings offer a new perspective for future explorations of circuit mechanisms mediating SCN functions, such as circadian rhythm, ingestive behavior, and osmotic stability. Therefore, it is critical to gather further functional and behavioral data on SCN CCK neurons.

## Author contributions

X-SY and H-HW designed and performed the experiments, analyzed data, and wrote the paper. WX performed the experiments. LW analyzed data and wrote the paper. W-MQ designed the experiments and analyzed data. R-XL and Z-LH designed the experiments, analyzed data, and wrote the paper.

### Conflict of interest statement

The authors declare that the research was conducted in the absence of any commercial or financial relationships that could be construed as a potential conflict of interest. The reviewer AH and the handling Editor declared their shared affiliation, at the time of review.

## Abbreviations

AH: anterior hypothalamic area
AI: agranular insular cortex
Arc: arcuate nucleus of the hypothalamus
BNST: bed nucleus of stria terminalis
Cg: cingulate cortex
DMH: dorsomedial nucleus of the hypothalamus
DR: dorsal raphe nucleus
IGL: intergeniculate leaf
LC: locus coeruleus
LH: lateral hypothalamic nucleus
LPO: lateral preoptic nucleus
LS: lateral septum
MnR: median raphe nucleus
MPO: medial preoptic nucleus
MO: motor cortex
OFC: orbital frontal cortex
PAG: periaqueductal gray
Prl: prelimbic cortex
PVH: paraventricular nucleus of the hypothalamus
PVT: paraventricular nucleus of the thalamus
RCh: retrochiasmatic area
SCN: suprachiasmatic nucleus
SON: supraoptic nucleus
SFO: subfomical organ
TC: tuber cinereum area
VDB: nucleus of the vertical limb of the diagonal band
VMH: ventromedial nucleus of the hypothalamus
VMPO: ventromedial preoptic nucleus
ZI: zona incerta.
