# Supplementary material for: Whole-Brain Monosynaptic Afferent Projections to the Cholecystokinin Neurons of the Suprachiasmatic Nucleus
Source: Front Neurosci. 2018 Nov 5;12:807. doi: 10.3389/fnins.2018.00807 (PMC6230653; doi:10.3389/fnins.2018.00807)
Supplement: Supplementary file 1 [file Data_Sheet_1.PDF]

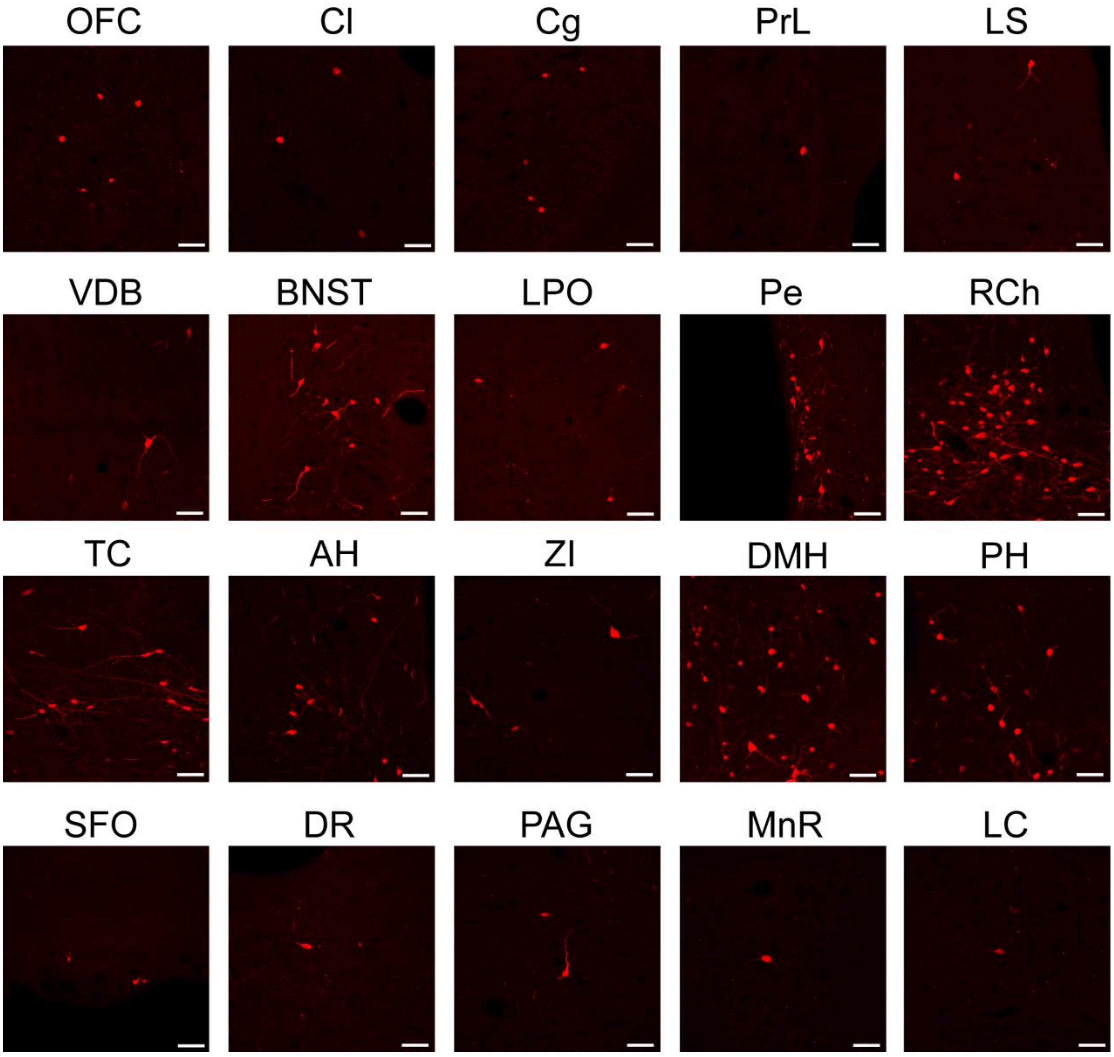

Supplemental figure 1 High magnification figure of with monosynaptic inputs to SCN CCK neurons. Scale bar, 50 μm. Data were obtained from four independent experiments. Abbreviations of the brain regions used are the following: AH, anterior hypothalamic area; BNST, bed nucleus of stria terminalis; Cg, cingulate cortex; Cl, claustrum; DMH, dorsomedial nucleus of the hypothalamus; DR, dorsal raphe nucleus; LC, locus coeruleus; LPO, lateral preoptic nucleus; LS, lateral septum; MnR, median raphe nucleus; PAG, periaqueductal gray; Pe, periventricular hypothalamic nucleus; PH, posterior hypothalamic area; PrL, prelimbic cortex; Rch, retrochiasmatic area; SFO, subfomical organ; TC, tuber cinereum area; VDB, nucleus of the vertical limb of the diagonal band; ZI, zona incerta.
